# Starvation at birth impairs germ cell cyst breakdown and increases autophagy and apoptosis in mouse oocytes

**DOI:** 10.1038/cddis.2017.3

**Published:** 2017-02-09

**Authors:** Yong-Yong Wang, Yuan-Chao Sun, Xiao-Feng Sun, Shun-Feng Cheng, Bo Li, Xi-Feng Zhang, Massimo De Felici, Wei Shen

**Affiliations:** 1 College of Life Sciences, Qingdao Agricultural University, Qingdao 266109, China; 2 College of Animal Science and Technology, Institute of Reproductive Sciences, Qingdao Agricultural University, Qingdao 266109, China; 3 Chengguo Station of Animal Husbandry and Veterinary, Laizhou 261437, China; 4 Department of Biomedicine and Prevention, University of Rome Tor Vergata, Rome 00133, Italy

## Abstract

The female reproductive lifespan is largely determined by the size of primordial follicle pool, which is established following germ cell cyst breakdown around birth. Almost two-third of oocytes are lost during germ cell cysts breakdown, following autophagic and apoptosis mechanisms. To investigate a possible relationship between germ cell cyst breakdown and nutrition supply, we established a starvation model in mouse pups at birth and evaluated the dynamics of cyst breakdown during nutrient deprivation. Our results showed that after 36 h of starvation between 1.5 and 3 d.p.p., indicators of metabolism both at systemic and ovarian level were significantly altered and the germ cell cyst breakdown markedly decreased. We also found that markers of oxidative stress, autophagy and apoptosis were increased and higher number of oocytes in cyst showing autophagic markers and of TUNEL-positive oocytes and somatic cells were present in the ovaries of starved pups. Moreover, the proliferation of pre-granulosa cells and the expression of the oocyte-specific transcription factor Nobox were decreased in such ovaries. Finally, we observed that the ovaries of the starved pups could recover a normal number of follicles after about 3 weeks from re-feeding. In conclusion, these data indicate that nutrient deficiency at birth can generate a number of adaptive metabolic and oxidative responses in the ovaries causing increased apoptosis both in the somatic cells and oocyte and autophagy mainly in these latter and leading to a delay of germ cell cyst breakdown and follicle assembly.

Germ cell cysts refer to a cluster of interconnected germ cells resulting from incomplete mitotic cytokinesis.^1, 2, 3^ In the mouse, germ cells form cysts from 10.5 to 13.5 days post coitum (d.p.c.) during the period of the primordial germ cell (PGC) proliferation in both sexes.^4, 5^ In female mice, cysts partially fragment prior to meiosis and gradually decrease in oocyte number throughout 17.5 d.p.c. to 4.0 days post partum (d.p.p.).^6^ In *Drosophila*, the mechanisms of germ cell cyst formation and its role in oogenesis have been elucidated. At first, one germline stem cell undergoes four times mitosis to form a 16-cell cyst, only one of these develops into an oocyte while the other 15 cells act as nurse cells. Such cells provide nutrients, proteins, mRNAs and organelles for the developing oocyte, transport their contents into the oocyte and undergo programmed cell death (PCD).^7, 8^

Likewise in the mouse, some oocytes within a cyst appear to receive nutrients and organelles from neighboring oocytes fated to undergo death.^9, 10^ It is not clear, however, whether oocytes in cysts die during or after cyst breakdown. In one model, one oocyte of a cyst dies following apoptosis and the large cyst breaks into two smaller cysts; this is repeated until a few individual oocytes remain.^11^ However, another study showed that cyst breakdown and apoptosis do not precisely coincide indicating that apoptosis is unlikely to be the major cause of cyst breakdown and that most of the oocytes actually die after cyst breakdown.^6^ Oocyte death during the fetal and early postnatal period has been described to occur by multiple causes and processes including apoptosis and autophagy.^12, 13, 14, 15^ Several lines of evidence demonstrate that inducers of oxidative stress may act as signaling molecules in the pathway of apoptosis in several, if not all, cells and tissues.^16, 17^ Oxidative stress occurs when the normal cellular redox balance is disturbed, resulting in dysregulation of redox-regulated processes and/or oxidative damage to cellular structures.^18, 19^ As a matter of fact, oxidative stress can impair the ovary's function and normal development of follicles and has been associated with polycystic ovary syndrome (PCOS).^20, 21^ Actually, in rodent models, increased ROS levels induce rapid primordial follicle loss and apoptosis in antral follicles.^22^ Whatever the exact mechanisms of oocyte apoptosis, in the mouse, more than two-third of oocytes die before and a few days after birth. At this time, cyst disappearance is concomitant with the formation of the primordial follicles (PFs). Processes extending from somatic cells have been observed between oocytes suggesting that somatic cells may physically separate oocytes.^11^ Physiologically, the size of the PF pool will largely determine the reproductive lifespan of female mammals.

The processes of cyst breakdown and follicle assembly likely involve communication between oocytes and pre-granulosa cells that is mediated and regulated by several factors. As a matter of fact, a number of compounds can influence cyst breakdown and consequently the pool of PFs. *In vivo* and *in vitro* exposure of neonatal mouse ovaries to E2, progesterone or phytoestrogens (i.e., genistein), inhibits cyst breakdown and PF assembly.^23, 24^ Synthetic forms of estrogens, such as diethylstilbestrol (DES), bisphenol-A (BPA) and ethinyl estradiol (EE), also block cyst breakdown and alter PF formation.^25, 26, 27^ Disruption of Notch signaling by pharmacological inhibition, global deletion of *Notch2* or *Hes1* (a downstream mediator of Notch signaling) or conditional knockout of *Lfng* (a negative modulator of *Notch*) or *Hes1* in somatic cells, result in abnormal oocyte maturation and multi-oocyte follicles (MOFs).^28, 29, 30, 31^ Other signaling molecules and several growth factors appear to have a role in follicle assembly. Among these, the stem cell factor (SCF, also known as Kit ligand, KL)^32, 33, 34, 35^ and neurotrophins^36, 37, 38^ favor such process. Mutants for either bone morphogenetic protein 15 (BMP15) or growth differentiation factor 9 (GDF9), have ovaries with a significant proportion of MOFs.^39^ Both BMP15 and GDF9 are secreted by the oocyte early in ovarian differentiation, supporting the notion that signals from the oocyte to the granulosa cells are important for cyst breakdown and follicle formation. Mouse ovaries exposed to another TGF*β* family member, activin A, showed an increased number of PFs.^40^ Sustaining a positive role of activin in PF formation, more MOFs were observed in mice overexpressing the activin antagonist, inhibin B.^41^ In addition, activin subunit expression is reduced in neonatal mice treated with E2, which blocks cyst breakdown.^42^ Mutants of another activin antagonist, follistatin, also have defects in oocyte development including a delay in cyst breakdown and follicle formation,^43^ while FST288, the strongest activin-neutralizing isoform, impaired germ cell nest breakdown and primordial follicle assembly by inhibiting pre-granulosa cell proliferation.^44^ The last TGF*β* family member that has been implicated in follicle assembly is anti-Mullerian hormone (AMH). PF assembly is reduced in newborn rat ovaries treated with AMH while oocyte number is greater than controls.^45^ A study using mouse ovary organ culture implicates FSH in promoting cyst breakdown and PF formation.^46^ Finally, a study in the mouse ovary showed that cyst breakdown requires the activity of c-Jun N-terminal kinases (JNKs) that are likely necessary to downregulate E-cadherin expression in oocytes.^47^

A number of transcription factors appear also to be important for PF formation. Mutant mice for the gene encoding the aryl hydrocarbon receptor (AHR), a basic helix loop helix (bHLH) transcription factor, form follicles at a faster rate than normal.^48, 49^ Mice lacking the factor in germline alpha (*Figla*), also encoding a bHLH protein, begin to lose oocytes at birth and oocytes still present are not enclosed in PFs.^50^ Disruption of *Nobox* (an oocyte-specific homeobox gene), results in increased mouse oocyte loss and a delay in cyst breakdown.^51, 52^ Mice mutant for *Foxl2*, (a winged-helix forkhead transcription factor), are sterile with germ cell cysts that do not breakdown.^53^ Finally, siRNA knockdown of heterogeneous nuclear ribonucleoprotein K in rat ovary organ culture caused a block in cyst breakdown and follicle formation.^54^ Recent data showed that PCNA (proliferating cell nuclear antigen) can regulate primordial follicle assembly by promoting apoptosis of oocytes in fetal and neonatal mouse ovaries.^55^

Many animals alter their reproductive strategies in response to environmental stress. For example, in female *Drosophila* and *Caenorhabditis elegans*, starvation activates apoptotic checkpoints and autophagy in oogenesis and reduces the production of mature oocytes.^56, 57^ In the perilous hours immediately after birth, a newborn mammal must survive the sudden loss of food supply from its mother. Under normal circumstances, newborns increase a metabolic response to ward off starvation until feeding occurs. Under these conditions, autophagy may become essential for viability of various tissues.^58^ The observation that in mouse the most part of the cyst breakdown occurring from 2 to 5 d.p.p., parallels the time when fully nutrient supply is restored by milk feeding, prompted us to investigate the influence of starvation on this process and PF assembly.

## Results

### Oocytes in cysts show morphological and molecular markers of autophagy

**Figure 1:**
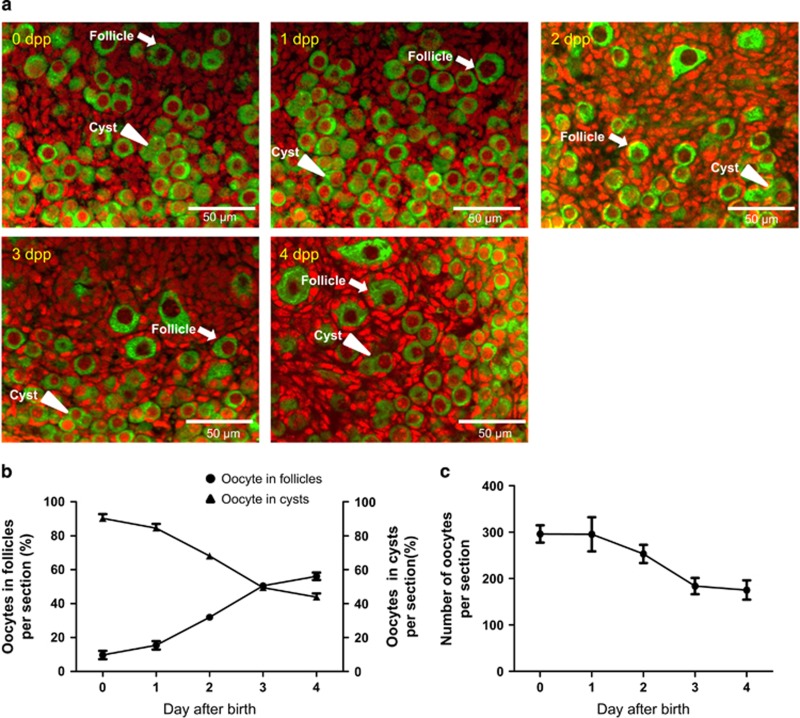
Dynamics of germ cell cyst breakdown. (**a**) Representative IF for oocytes with MVH (green) in tissue sections of ovaries at 0–4 d.p.p. Plenty of oocytes remaining within cysts (arrowheads) at 0–1 d.p.p., and quantification of oocytes were surrounded by pre-granulosa cells and formed primordial follicles (arrows) at 3–4 d.p.p. Scale bars: 50 *μ*m. (**b**) Percent of oocytes in follicles through 0 to 4 d.p.p.; note acceleration of cyst breakdown between 1 and 3 d.p.p., and quantification of oocyte number at 0–4 d.p.p.; note a marked decrease of the oocyte number between 1 and 3 d.p.p. (**c**) Number of oocytes per section in mouse ovaries of 0 to 4 d.p.p. Results are presented as mean±S.D. All the experiments were repeated at least three times

In a first series of experiments, we characterize the dynamics of germ cell cyst breakdown in mouse ovaries from 0 to 4 d.p.p. Immunolocalization of the oocytes with the germ cell specific marker MVH (mouse Vasa homolog) in tissue sections, revealed that while almost all oocytes were located in germ cell cysts (indicated by arrowheads) at 0 d.p.p., more than half of them were individually enclosed into follicles (indicated by arrows) at 4 d.p.p. (Figures 1a and b). Between 1 and 3 d.p.p., germ cell cyst breakdown was intense and associated with a major loss of oocytes (Figures 1b and c).

**Figure 2:**
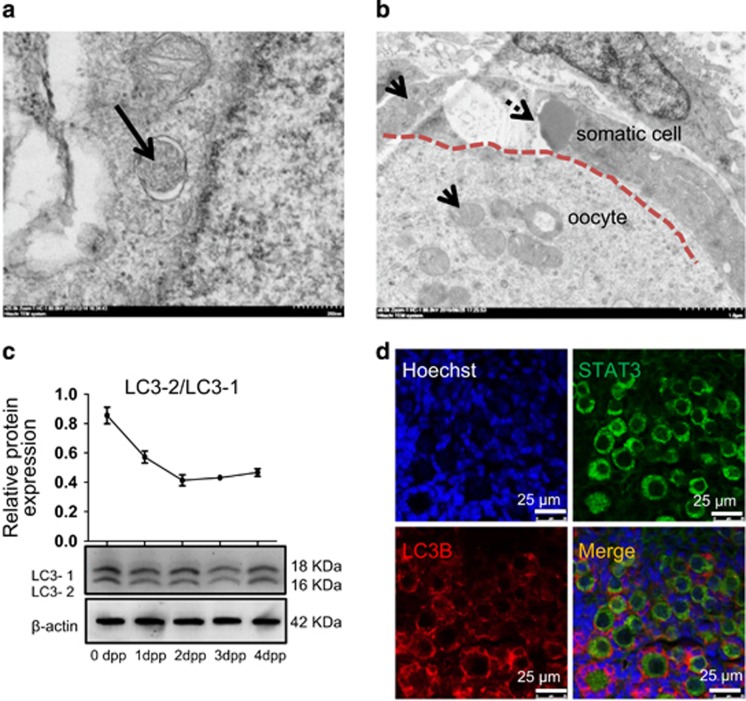
Morphological characteristics and autophagic markers in ovaries and oocytes at 0–4 d.p.p. (**a**) An autophagosome (arrow) in the oocyte cytoplasm. TEM, scale bar: 200 nm. (**b**) Numerous mitochondria (arrows) in the oocyte cytoplasm. Lipid droplets (arrows) in the cytoplasm of a somatic cell around oocytes. TEM, scale bars: 1 *μ*m. (**c**) WB for LC3-1 to LC3-2 in ovaries of 0–4 d.p.p. During autophagy, cytosolic LC3-1 is conjugated to phosphatidylethanolamine to form LC3-phosphatidylethanolamine conjugate (LC3-2), which is recruited to autophagosomal membranes. (**d**) IF for LC3B (red) and STAT3 (green) in 3 d.p.p. ovaries

TEM observations showed a frequent presence of autophagosomes, recognized for their characteristic spherical vesicle with double-layer membranes, suggesting macroautophagy, in the oocyte cytoplasm and of large number of mitochondria in some oocytes within cysts at 3 d.p.p. (Figure 2a, Supplementary Figures S1A and B). On the other hand, the somatic cells surrounding the oocytes showed less numerous mitochondria and smaller size lipid droplets often closely surrounded by mitochondria (Figure 2b). The expression of the autophagosome membrane-associated light chain 3-2 protein (LC3-2), in protein extracts from 0 to 4 d.p.p. ovaries was indicative of ongoing autophagy, while the relatively rapid decrease of the value of the ratio LC3-2/LC3-1 (the LC3 cytosolic form) from 0 to 3–4 d.p.p. suggested higher level of autophagy at the beginning of cyst breakdown (Figure 2c). Moreover, double IF staining for LC3B and the oocyte marker STAT3 of tissue sections of 3 d.p.p. ovaries revealed that most of the autophagic protein was localized in oocytes within cysts, primordial follicles and the granular cells of primary follicles (Figure 2d, Supplementary Figures S1C and D).

### Pup starvation for 36 h impairs primordial follicle assembly

**Figure 3:**
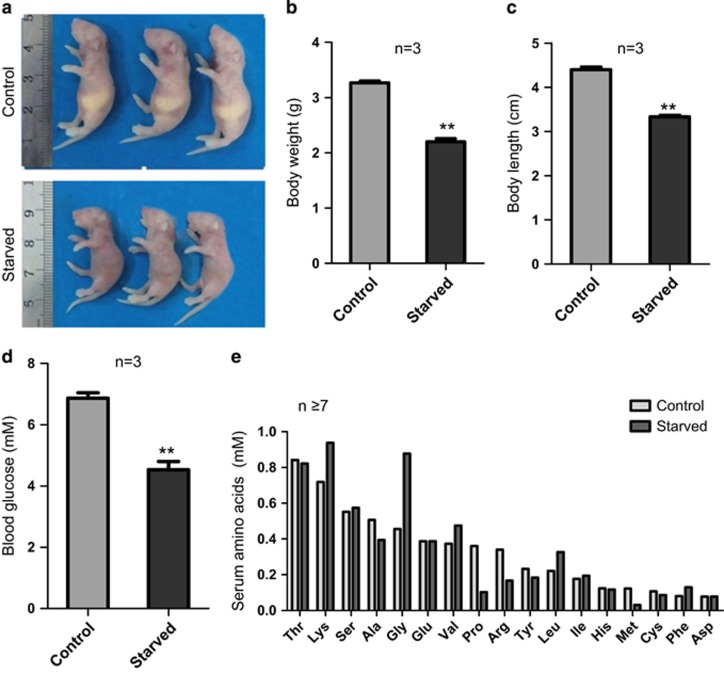
Systemic effects of pups starvation. (**a**) Photograph of starved pups compared with normal littermate. (**b** and **c**) Body weight and length of starved and control pups (*n*=3). (**d**) Concentration of blood glucose in starved and control pups (*n*=3). (**e**) Concentrations of 17 amino acids in the serum of starved and control pups (*n*=13). The results are presented as mean±S.D. *P<0.05; **P<0.01

To investigate whether nutrient supply can affect germ cell cyst breakdown and PF assembly, we first established a starvation model by depriving pups of nutrients for 36 h from 1.5 d.p.p. to 3 d.p.p. We observed that besides the overall physical appearance of emaciation (Figure 3a), the weight and length of the body of starved mice were significantly decreased in comparison with controls (*P*<0.01; Figures 3b and c). Furthermore, in mice of the experimental group, the concentration of glucose in the blood sharply lowered (*P*<0.01), while the amino acid metabolism appeared altered (Figures 3d and e).

**Figure 4:**
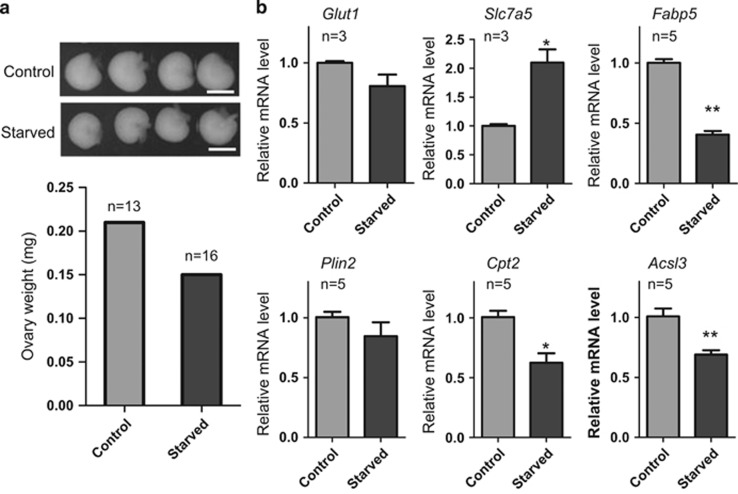
Alteration of size and transcript level of genes involved in metabolic pathways of starved pup ovaries. (**a**) Top: photograph of ovaries of starved and control pups. Scale bars: 500 *μ*m. Bottom: weight of ovaries from starved and control pups. (**b**) Quantitative RT-PCR of mRNA of the metabolism relevant genes *Glut1, Slc7a5, Plin2, Fabp5, Cpt2* and *Acsl3.* The results are presented as mean±S.D. All the experiments were repeated at least three times. *P<0.05; **P<0.01.

In the ovaries of the starved pups, the size and the transcript level of genes involved in metabolic pathways resulted also clearly altered. As a matter of fact, the ovaries of these pups were smaller and weighted significantly lesser than control (Figure 4a). Moreover, the lack of alteration of the mRNA level of *Glut1*, a gene encoding a major glucose transporter (Figure 4b), the decreased levels of transcripts of three genes encoding proteins of fatty acid synthesis such as *Fabp5*, *Cpt2* and *Acsl3,* although not of *Plin2* (*P*<0.05 or *P*<0.01; Figure 4b, Supplementary Figure S4), and the increased level of mRNA of *Slc7a5*, encoding a transporter of large neutral amino acids (*P*<0.01; Figure 4b), suggested compensatory changes in critical physiologies and defects in energy allocation, storage or utilization.

**Figure 5:**
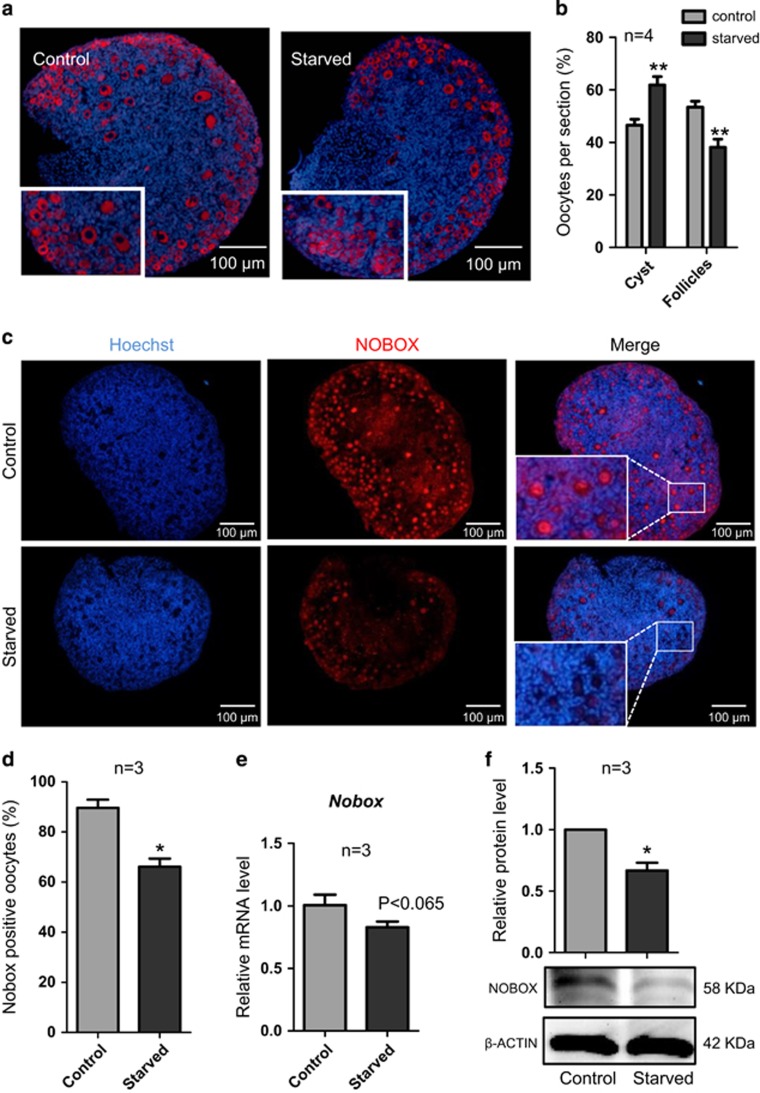
Pup starvation impairs germ cell cyst breakdown and follicle assembly. (**a**) Representative IF for the germ cell marker MVH in tissue sections of 3 d.p.p. ovaries from control and starved pups. Note that while in the control ovary, the most part of oocytes are formed cortical and medullary PFs, those in the starved ovary remained in cortical cysts. Scale bars: 100 *μ*m. (**b**) Quantification of the number of oocytes in cysts and in follicles in 3 d.p.p. control and starved ovaries. (**c**) Representative IF for the oocyte-specific transcription factor NOBOX in tissue sections of 3 d.p.p. ovaries from control and starved pups. Scale bars: 100 *μ*m. (**d**) Percentage of oocyte showing strong NOBOX staining in ovary tissue sections. (**e**) Quantitative RT-PCR for *Nobox* mRNA levels in control and starved ovaries. (**f**) Level of NOBOX protein in control and starved ovaries. The results are presented as mean±S.D. All the experiments were repeated at least three times. *P<0.05; **P<0.01

By counting the number of oocytes in cysts or enveloped individually into follicles in tissue sections of ovaries at 3 d.p.p., we found that the normal dynamics of cyst breakdown and PF formation was significantly impaired in starved pups (Figure 5a). Actually, in the ovaries of such group, the number of oocytes in cysts was higher and that in follicles lower in comparison with control, although the total number of oocyte did not change (Figure 5b).

### Markers of oxidative stress, autophagy and apoptosis are increased in ovaries of starved pups

**Figure 6:**
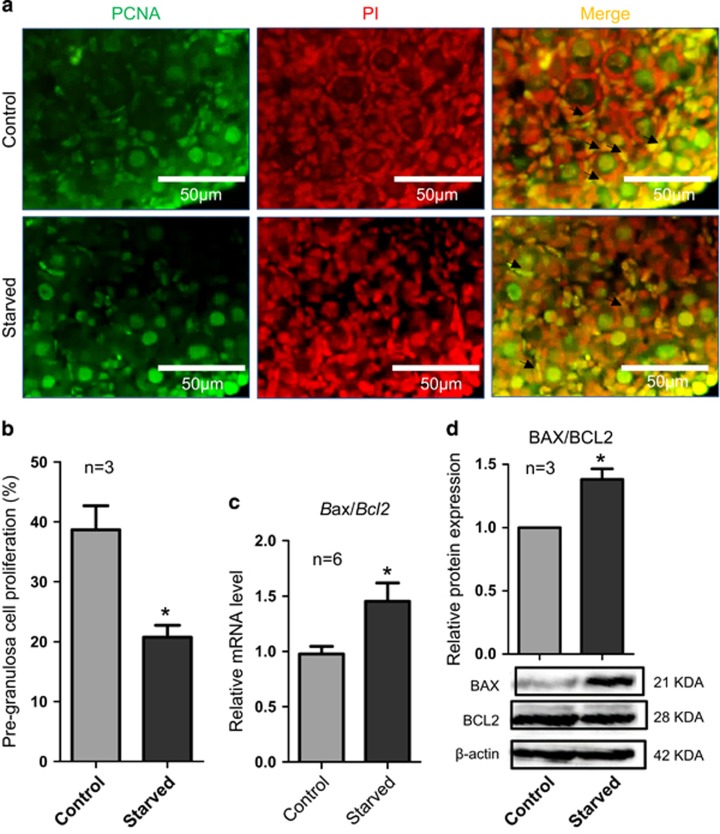
Proliferation and apoptotic markers in control *versus* starved ovaries. (**a**) PCNA histochemistry (green) in representative tissue sections of ovaries from normal and starved pups. (**b**) Percentage of relative pre-granulosa cell proliferation: PCNA-positive pre-granulosa cells/total oocytes in one section. (**c**) Quantitative RT-PCR for apoptosis relevant genes *Bax/Bcl2*. (**d**) Increased BAX/BCL2 protein ratio in starved ovaries in comparison with control. The results are presented as mean±S.D. All the experiments were repeated at least three times. *P<0.05; **P<0.01

To determine whether the defects in cyst breakdown and follicle assembly occurring in the ovaries of starved animals were associated to changes in processes such as oxidative stress, autophagy and apoptosis, we analyzed the mRNA level and/or proteins of the anti-oxidative enzymes *GPX1, SOD1*, *CLRX1* and *TXNRD1*, of apoptotic *Bax* and major proteins such as BCL2 and BAX and of the autophagic proteins LC3-2 and BECLIN1. Quantitative real-time PCR showed significant (*P*<0.05) increased transcripts of *Sod1*, *Clrx1* and *Txnrd1* and of the *Bax/Bcl2* mRNA ratio in starved ovaries (Figure 6a, Supplementary Figure S2). This later was confirmed also at protein level (Figure 6b). About the autophagic proteins, the number of oocytes in cysts showing spots of IF LC3-2 positivity was higher in starved than in control ovaries (Supplementary Figure S3A). Moreover, the level of BECLIN1 protein, evaluated by western blot (WB), decreased in ovaries after 12 h but returned to the control level after 24–36 h of starvation (Supplementary Figure S3B). Compared with control group, the ratio of Bax/Bcl2 in starvation group was increased in both the gene and protein levels. Surprisingly, number of TUNEL-positive cells was really small, there were rare cells found to be positive for apoptotic markers in both the starvation group and control group, especially in the control group (Figures 6c and d; Supplementary Figure S3C). And similar results could be found in previous publications.^11, 14^

At the same time, the number of pre-granulosa cells positive for the nuclear PCNA staining, generally associated to cell proliferation, was significantly reduced in starved in comparison with normal ovaries (*P*<0.05; Figures 6a and b). Moreover, among four oocyte-specific transcription factors analyzed such as *Lhx8*, *Sohlh2*, *Figlα* and *Nobox*, only the latter one resulted significantly reduced at protein level (Figures 5c–f and Supplementary Figure S2).

### Reversibility of the starvation effect on the ovarian follicle pool

**Figure 7:**
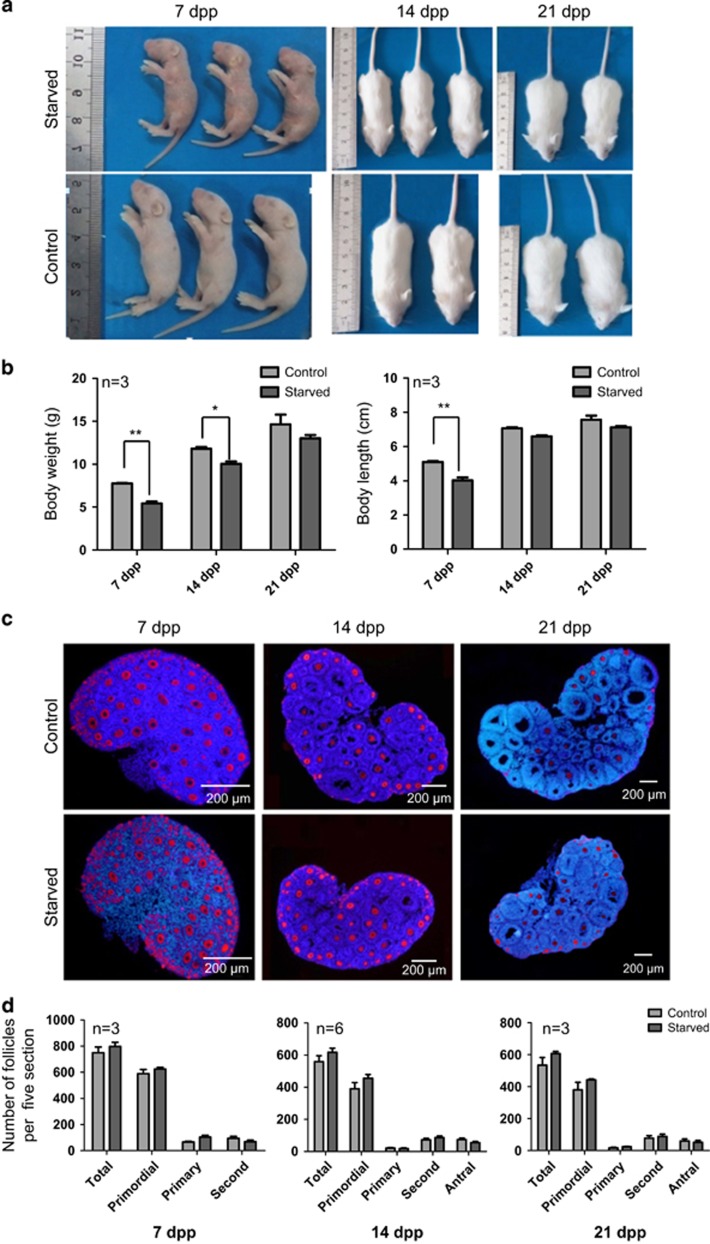
Reversibility of the starvation effect on the body size and ovarian follicle pool. (**a**) Starved pups compared with normal littermate after standard diet restoring. (**b**) Body weight and length of starved and control pups (*n*=3). (**c**) Representative IF for the germ cell marker MVH in tissue sections of control and starved ovaries after 7 d.p.p., 14 d.p.p. and 21 d.p.p. from standard diet restoring. (**d**) Quantification of the number of primordial, primary, secondary and antral follicles in control and starved ovaries after 7 d.p.p., 14 d.p.p. and 21 d.p.p. from standard diet restoring. All the experiments were repeated at least three times. *P<0.05; **P<0.01

We finally verified whether the ovaries of pups re-feeding after starvation were able to recover a normal PF pool. In this regard, we observed that after re-feeding, pups regained body weight and length, and at 21 d.p.p. (18 days from starvation), no significant difference remained between the control and starved animals (Figures 7a and b). At this time, the number of PF and of other stages as well, in the starved and normal ovaries were comparable (Figures 7c and d).

## Discussion

The results of our first analyses concerning the dynamics of germ cell cyst breakdown, showed that a peak in this process occurred between 1 and 3 d.p.p. and was associated to a marked decrease of the oocyte number. Moreover, morphological and molecular markers of autophagy were found in some oocytes inside cysts. All together, these data support the existence of a relationship between cyst breakdown and oocyte degeneration but do not clarify whether oocyte death precedes, is concomitant or occurs after such process. At the same time, the presence of autophagic markers in oocytes within cysts, primordial follicles and the granulosa cells of primary follicles, but not inside primary follicles, suggests that, under normal condition, activation in one or a few cystic oocytes of autophagy perhaps related to a large number of mitochondria observed at TEM, can be a prerequisite for their survival after cyst breakdown. In line with our observations, some of the mouse oocytes in cysts have been reported to acquire many organelles, including mitochondria, and other cytoplasmic components before primordial follicle formation.^10^ These materials, derived by transfer from interconnected oocytes, could be used as material for autophagy. Under starvation condition, autophagy could be exacerbated and ultimately lead to apoptosis. Actually, several evidences are accumulating that autophagy and apoptosis may be triggered by common upstream signals, sometimes converging in combined autophagy and apoptosis. The alteration of metabolic parameters and the increased expression of a number of enzymes involved in oxidative stress alongside with enhance of both autophagic and apoptosis markers in the ovaries from starved pups, testify a general cellular stress induced by the nutrient deprivation as the trigger of the cell death processes. Under such conditions, both pre-granulosa cell proliferation and expression of some oocyte transcription factors (i.e., Nobox), crucial for follicle assembly, appeared also to be impaired. This might explain the reduced rate of cyst breakdown. As *Nobox* is expressed mostly in primary (growing) oocytes and no change in transcripts level of other oocyte-specific genes such as *Lhx8*, *Sohlh2*, *Figlα* occur, oocyte growth could be also impaired in starved ovaries. Interestingly, the adverse effects of starvation on the ovaries appeared reversible. Actually, after about 3 weeks from pup re-feeding, the ovaries regained a normal number of follicles. This could indicate that cyst breakdown was only delayed and markers of oxidative stress, autophagy and apoptosis, increased in the starved ovaries. Despite these, the numbers of the remaining pre-granulosa cells and oocytes in such ovaries were apparently still sufficient to sustain the assembly of a normal number of follicles. Alternatively, this finding can be explained by the presence of ovarian or extra-ovarian stem cells able to sustain the ovary recovery.^59, 60^

Dehydration might be a non-negligible factor in ovary molecular changes even though the starved pups rejected to suck water and the starvation time was not so long. The main objective of this investigation was to explore the effects of starvation and the influence of dehydration on mouse primordial follicles formation would be the next phase study.^58, 61^

In conclusion, the present data indicate that nutrient deficiency at birth can generate a number of adaptive metabolic and oxidative responses in the ovaries causing increased apoptosis both in the somatic cells and oocyte and autophagy mainly in these latter and leading to impaired formation of the PF pool. Under the relatively short starvation time used by us (36 h), possibly thanks to such adaptive response, the ovary maintains the capability of regaining a normal follicle pool.

## Materials and methods

### Animals

CD1 mice were obtained from Vital River Laboratory Animal Technology Co. LTD (Beijing, China) and maintained in the animal room of Qingdao Agriculture University, *ad* *libitum*. Females were mated with males and the presence of the vaginal plug the morning after breeding, was considered as 0.5 day of pregnancy. For starvation protocols, pups were separated from mother at 1.5 d.p.p. for 36 h. To make sure to use pups that have received the same nutrition level before starvation, only animals with the same body weight (2.2±0.1 g) were selected. All procedures involving animals were approved by the Institutional Animal Care and Use Committees of Qingdao Agricultural University.

### Immunofluorescence

Ovaries were dissected and immediately fixed in 4% paraformaldehyde overnight. The samples were processed following standard histological procedures for paraffin embedding and serially sectioned at 5 *μ*m. The sections were heated at 60 °C in an air oven for 2 h, then immediately washed in xylene and rehydrated through a graded series of ethanol and soaked in PBS. Finally, they were transferred in 0.01 M sodium citrate buffer at high temperature (95 °C) for 10 min. After 1 h blocking with BDT (3% BSA, 10% normal goat serum in TBS), the sections were incubated with primary antibodies (Supplementary Table S2) in a humidified atmosphere overnight at 4 °C. Cy3-labeled goat anti-rabbit or FITC-conjugated goat anti-mouse secondary antibodies were used at dilution of 1 : 150 (Beyotime, A0516, A0568, Nantong, China) for 30 min at 37 °C in the dark. Counterstaining was performed with Hoechst33342 (Beyotime, C1022) or PI (Abcam, Cambridge, UK, ab14083). Oocytes in cysts or into primordial follicles were scored in every third section as previously described.^62, 63^

### TUNEL assay

TUNEL-positive cells were detected in paraffin sections of ovaries using the Bright Red Apoptosis Detect Kit (Vazyme, A113-02, Nanjing, China). Briefly, the sections were heated at the 60 °C in an air oven for 2 h, then immediately washed in xylene and rehydration through a graded series of ethanol, and soaked in PBS. The samples were then treated with proteinase K for 15 min at room temperature, rinsed twice with PBS and incubated for 60 min at 37 °C in the dark in 100 *μ*l of the TUNEL reaction mixture. Counterstaining was performed with Hoechst33342 (Beyotime, C1022).

### Transmission electron microscopy

The ovaries were dissected and immediately fixed in 2.5% glutaraldehyde in 0.2 M PBS (pH=7.2) overnight at 4 °C. The samples were processed and included in epoxypropane resin following standard transmission electron microscopy (TEM) procedures. Serial sections were cut at 50 nm using the EM UC7 ultramicrotome (Leica, Germany), stained with lead citrate and uranium and observed under HT7700 transmission electron microscope.

### WB analysis

Protein extracts were obtained from six ovaries and using the Cell Lysis Buffer for WB (Beyotime, P0013). The proteins were separated on 10% SDS-PAGE gel and transferred onto Immobilon-PSQ Transfer Membrane (Millipore MA, USA). After blocking, the membranes were incubated with the appropriate primary antibody (Supplementary Table S2) overnight at 4 °C. After washing three times in Tris-buffered saline and Tween 20 (TBST), the membranes were incubated at 37 °C for 2 h with horseradish peroxidase (HRP)-conjugated goat anti-rabbit (Beyotime, A0258) IgG or goat anti-mouse (Beyotime, A0216) IgG at 1 : 2000 dilution in TBST. Finally, the membranes were reacted with BeyoECL Plus Kit (Beyotime, P0018). *β*-Actin was used as housekeeping protein control.

### RNA extraction and quantitative real-time PCR

The mRNA was retrieved from two ovaries using the RNA Prep Pure Micro Kit (Aidlab RN07, Beijing, China), according to the manufacturer's descriptions and then reverse-transcribed into cDNA using TransScript One-Step gDNA Removal and cDNA Synthesis SuperMix (TransGen Biotech AT311-03, Beijing, China). Thermal cycler program was set as 50 min at 42 °C, 65 °C for 15 min, and finally a cooling step at 4 °C. Quantitative PCR (Supplementary Table S1) was carried out with Light Cycler real-time PCR instrument (Roche, Basel, Switzerland, LC480) using a Light Cycler SYBR Green I Master (Roche, 04887352001). Gene expression changes were analyzed by the 2^−△△Ct^ method and normalized to *β*-actin

### Dosage of blood amino acids and glucose

Samples of blood were collected from the mouse tail vein. For amino acid dosage, serum was separated from blood and de-proteinized with sulfosalicylic acid. Free amino acids in the supernatants were measured using an automated amino acid analyser (Hitachi L8900, Tokyo, Japan). Glucose concentrations were determined by dropping blood samples onto an Accu-Chek Active test strips (Roche, Mannheim, Germany) and measuring by Accu-Chek Active Blood Glucose Meter (Roche GC0612179, Basel, Switzerland).

### Statistical analyses

*T*-test was used to assess the difference between two groups (normal distribution)^40, 47, 64, 65^ and one-way analysis of variance (ANOVA) for multiple comparison tests to analyze the effects of starvation on BECLIN1 protein level in Supplementary Figure S3B in the ovary. Statistical analysis of follicle number counts was performed using Prism 5.0 (GraphPad Software Inc., San Diego, CA, USA).
