# Supplementary material for: Starvation at birth impairs germ cell cyst breakdown and increases autophagy and apoptosis in mouse oocytes
Source: Cell Death Dis. 2017 Feb 9;8(2):e2613–. doi: 10.1038/cddis.2017.3 (PMC5386484; doi:10.1038/cddis.2017.3)
Supplement: Supplementary Tables [file cddis20173x1.docx]

**T****ABLE S1.** Primers Used for Quantitative RT-PCR

| **Genes** | **Genbanks** | **Forward primer sequences** | **Reverse primer sequences** | **Product**  **Length(bp)** |
| --- | --- | --- | --- | --- |
| *Glut1* | NM_011400.3 | CAGCTGTCGGGTATCAATGC | TCCAGCTCGCTCTACAACAA | 150 |
| *Slc7a5* | NM_011404.3 | GCTCCGATTCAAGAAGCCTG | CTTGGGCTTGTTCTTCCACC | 199 |
| *Fabp5* | NM_010634.3 | AGGATCTCGAAGGGAAGTGG | TCTTCACTGTGCTCTCGGTT | 171 |
| *Plin2* | NM_007408.3 | GTTTTGGGGATGGTGCAGTT | CCAGCCGTTCATAGTTGCTC | 169 |
| *Nobox* | NM_130869.3 | CTATCCTGACAGTGACAAACGCC | CACCCTCTCAGCACCCTCATTAT | 251 |
| *Lhx8* | NC_000069.5 | CAGTTCGCTCAGGACAACAA | CCTGCAGTTCTGAAACCACA | 105 |
| *Sohlh2* | NM_028937.3 | TCTCAGCCACATCACAGAGG | GGGGACGCGAGTCTTATACA | 199 |
| *Figla* | NM_012013.1 | ACAGAGCAGGAAGCCCAGTA | TGGGTAGCATTTCCCAAGAG | 225 |
| *Cpt2* | NM_009949.2 | CGCCCAGCTTCCATCTTTAC | CTGCCAGATACCGTAGAGCA | 184 |
| *Acsl3* | NM_001033606.2 | AGGCTGCTGGGTGGAAATAT | CCACCTTCCTCCCAGTTCTT | 230 |
| *Gpx1* | NM_008160.6 | GGAGAATGGCAAGAATGAAGAG | AGGAAGGTAAAGAGCGGGTG | 135 |
| *Sod1* | NM_011434.1 | GGGTTCCACGTCCATCAGTA | TTGCCCAGGTCTCCAACAT | 128 |
| *Glrx2* | NM_001038592.1 | ACAGCACATCGTCGTTTTGG | ACGGTTCTTTCCCCAGTCAT | 235 |
| *Txnrd1* | NM_001042513.1 | GTGCATCCTATGTCGCCTTG | TCTCCTCGCTGTTTGTGGAT | 246 |
| *Bax* | NM_007527 | ATGCGTCCAAGGAAGACTGAG | CCCCAGTTGAAGTTGCCATCAG | 162 |
| *Bcl-2* | NM_009741 | GCAGAGATGTCCAGTCAG | CACCGAACTCAAAGAAGG | 127 |
| *Actin* | NM_007393.3 | TCGTGGGCCGCTCTAGGCAC | TGGCCTTAGGGTTCAGGGGGG | 255 |

**TABLE S2.** Antibodies

| **Antibody** | **Vendor** | **Dilution** |
| --- | --- | --- |
| MVH(IHC)  MVH(Double IF) | Abcam (ab13840)  mAbcam (ab27591) | 1:200  1:200 |
| LC3B (WB) | Sigma (102M4778V) | 1:1000 |
| LC3B (IHC) | Abcam (ab51520) | 1:1000 |
| NOBOX (IHC and WB) | Abcam (ab41521) | 1:1000(WB)/1:200(IHC) |
| BAX (WB) | Cell Signaling (#2772S) | 1:1000 |
| BCL-2 (WB) | Immuno Way (YT0470) | 1:1000 |
| PCNA (IHC) | ZSGB (ZM0213) | 1:200 |
| BECLIN1 (WB) | Cell Signaling (#3738) | 1:1000 |
| STAT3(IHC) | Cell Signaling(124H6) | 1:150 |
| β-ACTIN (WB) | Boster (BM0627) | 1:1000 |
